# Effect of ventricular volume on cerebrospinal fluid Alzheimer's disease biomarkers in patients with idiopathic normal pressure hydrocephalus

**DOI:** 10.1177/13872877251329081

**Published:** 2025-03-28

**Authors:** Aleksi Vanninen, Lauri Erkkilä, Tarja Kokkola, Tuomas Selander, Anne M Koivisto, Merja Kokki, Tadeusz Musialowicz, Anssi Lipponen, Mikko Hiltunen, Juhana Hakumäki, Sanna-Kaisa Herukka, Tuomas Rauramaa, Ville Leinonen

**Affiliations:** 1 Department of Neurosurgery, Kuopio University Hospital, Kuopio, Finland; 2 Neurosurgery, Institute of Clinical Medicine, University of Eastern Finland, Kuopio, Finland; 3 Institute of Clinical Medicine, University of Eastern Finland, Kuopio, Finland; 4 Department of Clinical Radiology, Diagnostic Imaging Center, Kuopio University Hospital, Kuopio, Finland; 5 Neurology, Institute of Clinical Medicine, University of Eastern Finland, Kuopio, Finland; 6 Science Service Center, Kuopio University Hospital (KUH) and School of Medicine, University of Eastern Finland (UEF), Kuopio, Finland; 7 NeuroCenter, Kuopio University Hospital, Kuopio, Finland; 8 Department of Neurosciences, University of Helsinki, Helsinki, Finland; 9 Department of Geriatrics, Helsinki University Hospital, Helsinki, Finland; 10 School of Medicine, University of Eastern Finland, Kuopio, Finland; 11 Department of Anaesthesia and Intensive Care Medicine, Kuopio University Hospital, Kuopio, Finland; 12 Institute of Biomedicine, University of Eastern Finland, Kuopio, Finland; 13 Department of Neurology, Kuopio University Hospital, Kuopio, Finland; 14 Department of Pathology, Kuopio University Hospital, Kuopio, Finland; 15 Pathology, Institute of Clinical Medicine, University of Eastern Finland, Kuopio, Finland

**Keywords:** Alzheimer's disease, brain, cerebrospinal fluid, cerebral ventricle, needle biopsy, normal pressure hydrocephalus

## Abstract

**Background:**

Idiopathic normal pressure hydrocephalus (iNPH) is a common disorder in aging populations. Alzheimer's disease (AD) is a significant comorbidity in iNPH patients, and the presence of AD pathology is associated with worse shunting outcomes. Cerebrospinal fluid (CSF) concentrations of AD-associated biomarkers in iNPH patients are universally reduced and the exact mechanism related to this is unknown.

**Objective:**

Our aim was to study the effects of ventricular volume on CSF AD-associated biomarker levels in iNPH patients, to determine whether a dilution effect occurs and to assess if brain AD pathology contributes to this effect.

**Methods:**

A total of 153 iNPH patients had lumbar CSF samples available for analysis, along with brain MRIs of sufficient quality. Automated image analysis software was used to determine the volume of different brain segments. Volumes normalized for age, sex and head size were used for analysis. Brain biopsy data on AD pathology was also available.

**Results:**

None of the intracerebral ventricular volumes correlated with CSF levels of AD-associated biomarkers, indicating no dilution effect was present in this context. However, in iNPH patients positive for amyloid-β pathology in the biopsy, the volume of the fourth ventricle correlated inversely with all investigated biomarkers.

**Conclusions:**

Intracerebral ventricular volumes do not correlate with AD biomarker levels in CSF, arguing against a dilution effect. However, in patients with AD pathology, the volume of the fourth ventricle is inversely correlated with CSF T-Tau and P-Tau_181_ levels, suggesting a complex relationship between brain AD pathology, CSF flow and CSF volume in iNPH patients.

## Introduction

Idiopathic normal pressure hydrocephalus (iNPH) is a progressive neurological disorder characterized by the triad of impaired gait, urinary incontinence, and mild cognitive impairment.^1^ The radiological hallmark of iNPH is a disproportionally enlarged subarachnoid space, presenting as enlarged ventricular spaces, a narrowed superior convexity subarachnoid space and often enlarged sylvian fissures.^2^ INPH is more prevalent in the aging population, with a reported prevalence of up to 8.9% in patients over 80 years old.^3,4^ Shunt surgery improves the symptoms of iNPH, and earlier surgical intervention increases survival rates.^5,6^ A significant proportion of patients with shunted iNPH develop Alzheimer's disease (AD) during follow-up, leading to worse long-term cognitive outcomes.^7,8^

AD is the most common cause of dementia worldwide and is associated with significant societal costs.^9^ The main theory on the pathogenesis of AD is the amyloid cascade hypothesis, which posits that amyloid plaques aggregate in brain tissue, leading to neuronal damage and the formation of tau aggregates ultimately resulting in clinical AD.^10^ New interventions targeting amyloid plaques have been developed to reverse amyloid pathology.^11,12^ Although current anti-amyloid treatments provide only moderate effects and are associated with significant adverse effects, they still represent progress in the development of AD therapies.^13^ Early detection of AD will be vital for initiating future treatments early enough to modify the disease course.^14,15^ Currently early detection of AD is important to address comorbid conditions associated with faster progression of AD such as hypertension and type 2 diabetes.^16^ Traditional biomarkers for AD include lumbar cerebrospinal fluid (CSF) amyloid-beta 1-42 (Aβ_1-42_), total tau protein (T-Tau) and phosphorylated tau (P-Tau_181_).^17^ The concentration of these markers chance as AD pathology develops.^18^

INPH appears to reduce the levels of CSF AD biomarkers across the board, complicating the diagnosis of AD in iNPH patients. Hypothesized causes for this reduction include decreased cortical metabolism, impaired CSF clearance or a simple dilution effect.^19,20^ However, a recent study contradicts the dilution theory.^21^ A cortical biopsy taken during the shunt procedure can aid in predicting the development of AD post-surgery, though it does not help with preoperative diagnostics.^7^ The presence of amyloid and tau pathology in the cortical biopsy correlates well with amyloid PET imaging findings.^22^

In this study, we aimed to investigate the relationship between CSF levels of AD biomarkers and different anatomical areas of the brain in patients with iNPH. Our hypothesis was that the CSF concentration of AD biomarkers would decrease as ventricular volume increases.

## Methods

### Patient cohort

**Figure 1.**
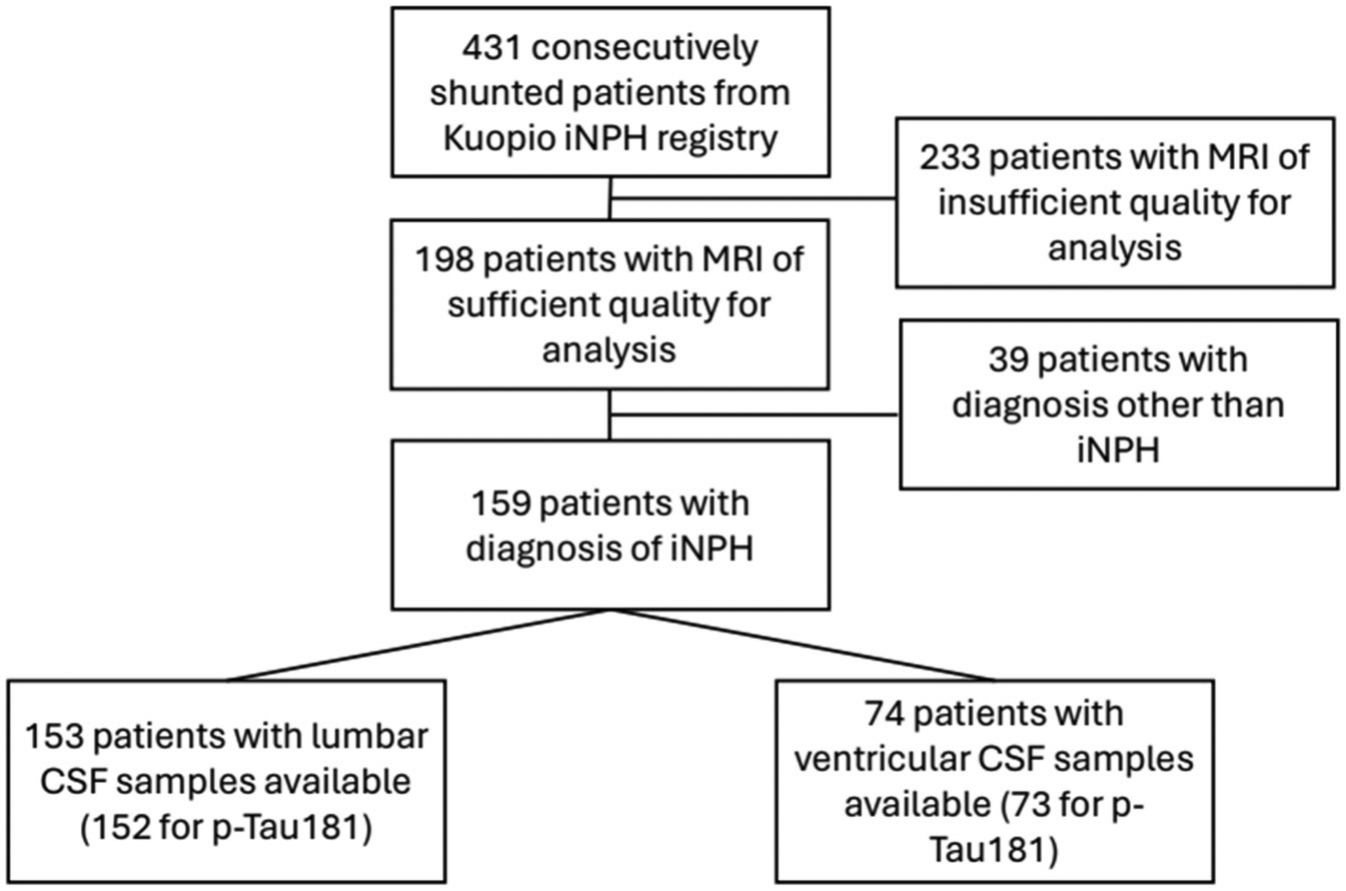
Patient cohort flowchart. iNPH: idiopathic normal pressure hydrocephalus; MRI: magnetic resonance imaging; CSF: cerebrospinal fluid; p-Tau181: Phosphorylated Tau 181.

Patients presenting with at least one of the three core symptoms of NPH (gait difficulty, mild cognitive impairment, or urinary incontinence) along with enlarged brain ventricles were evaluated at Kuopio University Hospital (KUH) neurosurgery. Patients were evaluated by a clinician, and those diagnosed with possible iNPH were included in the Kuopio NPH registry.^1,23^ The diagnosis of probable iNPH was made according to Relkin et al. (2005) criteria.^1^ Distinction between idiopathic and secondary NPH was made according to patient history. Shunt response was evaluated with gait speed reported at m/s and/or symptom improvement on iNPH grading scale.^23,24^ A total of 198 patients had magnetic resonance imaging (MRI) of sufficient quality for volumetric analysis. Out of these patients, 39 were determined to have secondary NPH or a different diagnosis altogether, such as long-standing overt ventriculomegaly in adults, and were excluded from the cohort. A total of 153 patients had lumbar CSF samples available for analysis (152 had P-Tau_181_ samples available). Ventricular CSF samples were available for 74 patients. The cohort is presented in Table 1A and 1B. A flowchart of our cohort is presented in Figure 1. Shunt response in this study is reported at 3 month follow up.

**Table 1A.** Patient demographics.

Patient demographics	All (N = 153)^,^^	Amyloid + (N = 77)	Amyloid – (N = 76)	p*	Tau + (N = 33)	Tau – (N = 121)	p*
Age^+^ (y), mean (SD)	75.0 (6.3)	77.6 (4.9)	72.3 (6.4)	<0.001	77.7 (5.6)	74.2 (6.3)	0.004
Preoperative BMI, mean (SD)	27.8 (5.2)	26.8 (4.8)	28.92 (5.6)	0.02	24.8 (4.3)	28.7 (5.2)	<0.01
Female sex; N (%)	80 (50.3%)	44 (57.1%)	30 (39.5%)	0.03	17 (51.5%)	58 (47.9%)	0.77
Amyloid pathology +, N (%)	77 (50.3%)	-	-	-	23 (69.7%)	54 (45.0%)	0.01
Tau pathology +, N (%)	33 (21.4%)	23 (29.9%)	10 (15.2%)	0.01	-	-	-
Shunt response, N (%)	118 (84.9%)	53 (68.8%)	59 (89.4%)	0.1	24 (82.8%)	89 (84.8%)	0.795
*APOE* ε4 carrier** N (%)	54 (34.0%)	39 (50.6%)	12 (15.8%)	<0.001	12 (36.4%)	39 (32.2%)	0.657

Amyloid +: Patients with amyloid pathology in brain biopsy; Amyloid –: Patients with no amyloid pathology in brain biopsy; Tau +: Patients with tau pathology in brain biopsy; Tau –: patient with no tau pathology in brain biopsy. ^N for *APOE* status was 159, ^^ N for shunt response (responder or non-responder) was 139, *p-values were determined using independent samples t-test. y: years; SD: standard deviation; N: total number of subjects, **all *APOE* ε4 carriers were heterozygous, + Age range for all subjects was 53.5–86.4 and 2 of patients shunted were under 60 years of age.

**Table 1B.** CSF biomarker levels in iNPH patients according to brain biopsy pathology.

Biomarkers	Type (N)	All patients	Amyloid +	Amyloid−	Tau + Amyloid +*	Tau + Amyloid−**
Aβ_1_**_-_**_42_ ng/l, mean (SD)	L-CSF (153)	762.7 (246.3)	683.7 (205.2)	833.0 (257.3)	629.2 (201.5)	745.9 (319.4)
	V-CSF (74)	449.1 (161.7)	424.3 (141.7)	466.2 (179.6)	532.4 (167.324)^×^	320.1 (133.2)
T-Tau ng/l, mean (SD)	L-CSF (153)	158.8 (49.6)	171.1 (49.4)	145.8 (48.0)	180.5 (43.5)^×^	139.8 (53.8)
	V-CSF (74)	305.7 (191.3)	336.7 (177.7)	272.8 (204.5)	451.6 (238.5)^×^	150.2 (38.6)
P-Tau_181_ ng/l, mean (SD)	L-CSF (152)	11.8 (5.1)	13.4 (5.4)	10.2 (4.4)	14.2 (4.4)^×^	9.7 (6.1)
	V-CSF (74)	13.8 (7.4)	16.4 (8.3)	11.2 (5.8)	22.8 (13.2)^×^	5.8 (2.9)

Amyloid +: Patients with amyloid pathology in brain biopsy; Amyloid –: Patients with no amyloid pathology in brain biopsy; Tau +: Patients with tau pathology in brain biopsy; Tau –: patient with no tau pathology in brain biopsy; SD: standard deviation; N: total number of subjects; Aβ_1-42_: CSF concentration of amyloid beta 1-42; L-CSF: lumbar cerebrospinal fluid; V-CSF: ventricular cerebrospinal fluid; T-Tau: CSF concentration of total tau protein; P-Tau_181_: CSF concentration of phosphorylated Tau 181. *N = 23, **N = 10, ^× ^= p < 0.05 versus Tau + Amyloid − -group.

### CSF samples

CSF samples were obtained from iNPH patients by lumbar puncture in outpatient clinic before 12 AM. Samples were obtained after a period of fasting. Samples were then centrifuged and stored at −80°C until analysis at the UEF biomarker laboratory, following a standardized protocol. Storage time for ventricular samples varied between 3 months to 6 years. For lumbar samples there was no significant storage time. Until 2020, AD biomarker levels (Aβ_1-42_ t-Tau, and P-Tau_181_) were analyzed using enzyme-linked immunosorbent assays by Innotest (Fujirebio, Ghent, Belgium). Since 2020, AD biomarkers have been measured with Elecsys assays (Roche Diagnostics, Penzberg, Germany). In 2022, the assay was changed to the gen2 Elecsys kit (Roche Diagnostics, Penzberg, Germany). Innotest kit levels were converted to Elecsys gen2 values using conversion equations featured in Table 2.^25^ Elecsys gen1 Aβ_1-42_ values were transformed into Elecsys gen 2 values using equation featured in Table 2. These conversions were validated by UEF biomarker laboratory and were similar to previously reported conversion factors.^26^

**Table 2.** Conversion equation for innotest and elecsys analysis methods.

Marker	Function
Aβ_1__-__42_	ElecsysGen2(Aβ_1-42_) = (0.955*Innotest[Aβ_1-42_]) + 125.6
Aβ_1-42_	ElecsysGen2(Aβ_1-42_) = (0.797*ElecsysGen1(Aβ_1-42_)) + 119.75
T-Tau	Elecsys(T-Tau) = (0.475*Innotest[T-Tau]) + 66.0
P-Tau_181_	Elecsys(P-Tau_181_) = (0.419*Innotest[P-Tau_181_]) – 3.87

Aβ_1-42_: amyloid-beta 1-42; t-Tau: total tau protein; P-Tau_181_: phosphorylated tau 181. Elecsys(x)=Elecsys value for biomarker x; Innotest[x] = Innotest value for biomarker x. ElecsysGen2(Aβ_1-42_) = Second generation Elecsys assay, which only affects amyloid levels.

### APOE genotyping

*APOE* genotype was determined from venous blood via PCR method as described previously.^27^

### Brain biopsy samples

The biopsy procedure has been described previously.^23^ Three cylindrical cortical biopsies, measuring 2—5 mm in diameter and 2–7 mm in length, were obtained using a TTI46 biopsy needle (Merit Medical Systems Inc. South Jordan, UT, USA) during shunt surgery. The samples were placed in buffered formalin and embedded in paraffin. For analysis, the paraffin-embedded samples were cut into 7-micron thick sections and stained with hematoxylin-eosin. The samples were then stained immunohistochemically using the following antibodies: (Tau (Clone; AT8, company; Innogenetics, Ghent, Belgium), Aβ ((Clone; 6F/3D, Company; Dako) and p62 (Clone; 3, Company; BD Biosciences Pharmingen, Franklin Lakes, NJ, USA). A neuropathologist assessed the samples under light microscopy, and the presence of amyloid and tau pathology was assessed semiquantitatively as either present or absent.^28^

### Imaging analysis

The patients underwent standard brain MRI scans with 3-D T1 image volumes on several 1.5 T (Siemens Healthineers, Erlangen, Germany) and 3.0 T (Philips, Best, The Netherlands) MRI machines prior to the shunt procedure.

MRIs were analyzed using cNeuro software (Combinostics Oy, Tampere, Finland). The Brain MRIs were automatically analyzed, and whole brain segmentation was performed.^29^ The accuracy of the automated analysis method was previously validated in the ADNI cohort.^30^ In our analysis, we used the volumetric segmentation of the ventricular spaces and different regions of the brain. Normalized values for the brain segment volumes were used in the analyses to reduce the effect of age, sex, and head size. Comparisons were also performed using absolute values. The interpretation of results was conducted according to normalized values.

### Statistical methods

SPSS Statistics (version 27.0 SPSS Inc., Chicago, IL, USA) was used for statistical analyses. Lumbar and ventricular CSF values for P-Tau_181_ were below the lower limit of quantification in 8 patients. Lumbar CSF (L-CSF) Aβ_1-42_ levels were above the upper limit of quantification in 5 patients. One patient had L-CSF Aβ_1-42_ levels below the lower limit of quantification, and one patient had ventricular CSF (V-CSF) Aβ_1-42_ levels below the lower limit of quantification. Additionally, the L-CSF T-Tau level was below the lower limit of quantification in one patient. Values below the lower limit of quantification were replaced with minimum values, while values above the upper limit of quantification were replaced with maximum values.

Spearman's correlation analysis was used to study associations between measurements. Variables with significant correlations (p-value < 0.05) were included in the linear regression analysis. Logarithmic transformation was applied to CSF biomarker values in linear regression to achieve a better fit to the normal distribution. The Stepwise method of variable selection was used for analysis when more than 10 variables showed significant correlation with the investigated CSF biomarkers. p-values <0.05 were considered statistically significant.

Linear regression was used to test associations between L-CSF AD biomarkers and ventricular volume in groups divided by amyloid and tau pathologies on brain biopsy. *APOE* ε4 carrier status and BMI were included as covariates in these analyses due to their potential effect on CSF biomarker levels.^31^

Independent samples T-test was used to compare subgroups.

## Results

The final regression models for the L-CSF biomarkers are presented in Table 3. Interestingly, the only ventricular space that was significant in the linear regression analysis was the 4th ventricle in relation to t-Tau. As the volume of the 4th ventricle increased the L-CSF t-tau levels decreased. The final regression models for the L-CSF biomarkers are presented in Table 3.

**Table 3.** Linear regression models for investigated L-CSF biomarkers in relation to regional brain volumes*.

Dependent	log_10_P-Tau_181_		
Variable	B	p	95% C.I.
Gyrus Rectus	0.196	0.007	0.054–0.339
Left superior parietal lobule	−0.044	<0.001	−0.069 – −0.019
Right occipital pole	0.083	0.005	0.025–0.142
Left posterior orbital gyrus	0.125	0.013	0.027–0.223
Total vessel volume	−1.853	0.022	−3.437 – −0.270

Dependent	log_10_T-Tau		
Variable	B	p	95% C.I.
Right Occipital pole	0.050	0.009	0.013–0.088
4^th^ ventricle	−0.035	0.002	−0.057 – −0.014
Superior temporal gyri	−0.016	0.024	−0.029 – −0.002
Left posterior orbital gyrus	0.091	0.005	0.028–0.154
Hippocampus	−0.038	0.011	−0.068 – −0.009

Dependent	log_10_Aβ_1-42_		
Variable	B	p	95% C.I.
Right superior occipital gyrus	0.066	0.014	0.014–0.119
Left transverse temporal gyrus	0.183	0.015	0.037–0.329

CSF: cerebrospinal fluid; *Only statistically significant variables are presented; B: Regression coefficient; C.I.: Confidence interval for regression coefficient; Aβ_1-42_: amyloid beta 1-42; t-Tau: total tau protein; P-Tau_181_: phosphorylated tau 181.

A larger hippocampal volume indicated lower observed levels of t-Tau in the linear regression analysis, but this finding was not observed with other imaging biomarkers.

Interestingly, when using non-normalized brain segment volumes for analysis, the volume of the 4th ventricle was significant in relation to all investigated L-CSF biomarker levels. This difference between analyses is most likely explained by the effect of age and head size on brain segment volume and CSF biomarker levels. The linear regression results for non-normalized values are presented in Supplemental Tables 1–3.

The presence of amyloid pathology in brain biopsy was statistically significant for both P-Tau_181_ and T-tau levels, with p-values of <0.001 and 0.002, respectively, with increased levels of tau proteins in patients with amyloid pathology.

In the linear regression analysis of patients with amyloid pathology, the volume of 4th ventricle showed significant inverse correlation with L-CSF T-Tau and P-Tau_181_. None of the ventricular spaces were statistically significant in patients without amyloid pathology. In patients without amyloid pathology in biopsy *APOE* ε4 had inverse correlation with Aβ_1-42_ and T-Tau L-CSF concentrations. The regression models are presented in Table 4.

**Table 4.** Linear regression models for relation between ventricle size and lumbar CSF biomarkers according to amyloid pathology.

Category				
Amyloid + Aβ_1-42_	Variable	Regression coefficient	p	95% C.I.
	3^rd^ Ventricle	−0.022	0.394	−0.074–0.029
	4^th^ Ventricle	−0.038	0.081	−0.081–0.005
	Lateral Ventricles	0.002	0.829	−0.012–0.013
	Total ventricular volume	−0.001	0.374	−0.015–0.013
	BMI	0.003	0.374	−0.004
	*APOE* ε4 carrier	−0.034	0.307	−0.100–0.032
Amyloid + T-Tau				
	3^rd^ Ventricle	−0.004	0.881	−0.057–0.049
	4^th^ Ventricle**	−0.074	0.001	−0.118 – −0.031
	Lateral Ventricles	−0.006	0.361	−0.020–0.008
	Total ventricular volume	0.006	0.353	−0.007–0.020
	BMI	−7.12*10^-5	0.984	-0.007–0.007
	*APOE* ε4 carrier	0.006	0.862	-0.061–0.072
Amyloid + P-Tau_181_				
	3^rd^ Ventricle	−0.008	0.884	−0.087–0.072
	4^th^ Ventricle*	−0.092	0.007	−0.157 – −0.027
	Lateral Ventricles	−0.006	0.566	−0.027–0.015
	Total ventricular volume	0.005	0.613	−0.016–0.026
	BMI	−0.003	0.635	−0.013–0.008
	*APOE* ε4 carrier	0.022	0.658	−0.078–0.123
Amyloid - Aβ_1-42_				
	3^rd^ Ventricle	0.022	0.491	−0.031–0.074
	4^th^ Ventricle	−0.016	0.651	−0.086–0.054
	Lateral Ventricles	0.009	0.482	−0.017–0.034
	Total ventricular volume	−0.009	0.482	−0.033–0.016
	BMI	−0.005	0.187	−0.012–0.002
	*APOE* ε4 carrier*	−0.145	0.014	−0.260 – −0.030
Amyloid - T-Tau				
	3^rd^ Ventricle	0.018	0.385	−0.023–0.060
	4^th^ Ventricle	−0.034	0.229	−0.089–0.022
	Lateral Ventricles	0.008	0.440	−0.012–0.013
	Total ventricular volume	−0.007	0.494	−0.026–0.006
	BMI	−0.003	0.239	−0.009–0.002
	*APOE* ε4 carrier*	−0.096	0.038	−0.186 – −0.006
Amyloid - P-Tau_181_				
	3^rd^ Ventricle	0.053	0.137	−0.017–0.112
	4^th^ Ventricle	−0.012	0.799	−0.104–0.081
	Lateral Ventricles	0.022	0.200	−0.012–0.055
	Total ventricular volume	−0.021	0.200	−0.053–0.011
	BMI	0.001	0.783	−0.008–0.011
	*APOE* ε4 carrier	−0.076	0.320	−0.227–0.075

Amyloid +: amyloid pathology in brain biopsy; Amyloid –: No amyloid pathology in brain biopsy; Aβ_1-42_: amyloid beta 1-42; t-Tau: total tau protein; P-Tau_181_: phosphorylated tau 181; C.I.: confidence interval; *Statistically significant at p < 0.05; **statistically significant at p < 0.005; *APOE* ε4 carrier: heterozygous *APOE* ε4 genotype; BMI: body mass index.

In patients with tau pathology, the volume of 4th ventricle was inversely correlated with P-Tau_181_ and T-Tau levels, but not with Aβ_1-42_-levels. In patients without tau pathology, T-Tau levels decreased significantly with larger 4th ventricle volumes. *APOE* ε4 status correlated inversely with L-CSF Aβ_1-42_ in patients without tau pathology in biopsy. Regression results are presented in Table 5.

**Table 5.** Linear regression models for relation between ventricle size and lumbar CSF biomarkers according to tau pathology.

Category				
Tau + Aβ_1-42_	Variable	Regression coefficient	p	95% C.I.
	3^rd^ Ventricle	−0.019	0.595	−0.092 −0.054
	4^th^ Ventricle	−0.055	0.236	−0.149–0.039
	Lateral Ventricles	0.005	0.549	−0.013–0.024
	Total ventricular volume	−0.005	0.582	−0.022–0.013
	BMI	−0.003	0.630	−0.016–0.010
	*APOE* ε4 carrier	−0.094	0.104	−0.209–0.021
Tau + T-Tau				
	3^rd^ Ventricle	0.024	0.464	−0.042–0.090
	4^th^ Ventricle*	−0.118	0.011	−0.205 – −0.030
	Lateral Ventricles	−0.016	0.060	−0.032–0.001
	Total ventricular volume	0.014	0.070	−0.001–0.030
	BMI	−0.001	0.852	−0.013–0.011
	*APOE* ε4 carrier	0.023	0.644	−0.079–0.126
Tau + P-Tau_181_				
	3^rd^ Ventricle	0.075	0.222	−0.048–0.198
	4^th^ Ventricle*	−0.163	0.049	−0.326 – −0.001
	Lateral Ventricles	−0.016	0.277	−0.046–0.014
	Total ventricular volume	0.014	0.333	−0.015–0.043
	BMI	−0.002	0.857	−0.023–0.020
	*APOE* ε4 carrier	0.063	0.497	−0.127–0.253
Tau - Aβ_1-42_				
	3^rd^ Ventricle	0.023	0.289	−0.020–0.066
	4^th^ Ventricle	−0.023	0.271	−0.065–0.019
	Lateral Ventricles	0.009	0.315	−0.008–0.026
	Total ventricular volume	−0.009	0.311	−0.026–0.008
	BMI	−0.003	0.290	−0.008–0.003
	*APOE* ε4 carrier**	−0.110	<0.001	−0.170 – −0.049
Tau - T-Tau				
	3^rd^ Ventricle	−0.017	0.384	−0.056–0.022
	4^th^ Ventricle*	−0.041	0.036	−0.079 – −0.003
	Lateral Ventricles	0.001	0.885	−0.015–0.017
	Total ventricular volume	0.000	0.953	−0.008–0.002
	BMI	−0.003	0.186	−0.008–0.002
	*APOE* ε4 carrier	0.012	0.417	−0.044–0.067
Tau - P-Tau_181_				
	3^rd^ Ventricle	−0.025	0.394	−0.084–0.034
	4^th^ Ventricle	−0.039	0.184	−0.097–0.019
	Lateral Ventricles	0.004	0.770	−0.020–0.027
	Total ventricular volume	−0.003	0.767	−0.027–0.020
	BMI	−0.002	0.643	−0.009–0.006
	*APOE* ε4 carrier	0.052	0.223	−0.032–0.135

Tau +: Tau pathology in brain biopsy; Tau –: No tau pathology brain biopsy; Aβ_1-42_: amyloid beta 1-42; t-Tau: total tau protein; P-Tau_181_: phosphorylated tau 181; C.I.: confidence interval; *Statistically significant at p < 0.05; **statistically significant at p < 0.005; *APOE* ε4 carrier: heterozygous *APOE* ε4 genotype: BMI: body mass index.

In our study population, there were 10 patients with tau pathology without amyloid pathology. These patients most likely have a comorbid neurological disorder other than AD. This group showed statistically significant differences in all tested biomarkers except for lumbar CSF Aβ_1-42_ against Tau + amyloid + -group. This is presented in Table 1B. Unexpectedly, the Tau + amyloid + group had higher ventricular CSF concentration of Aβ_1-42_ than Tau + amyloid – group. N for ventricular samples in these groups was low (N = 6 and N = 5 respectively) so this should be interpreted cautiously.

## Discussion

In this study, we investigated the relationship between brain areas and CSF concentrations of AD biomarkers, with a primary focus on the effect of ventricle size on biomarker concentrations. Our results support those of a previous study on intracerebral ventricular spaces.^21^ Ventricular volume did not seem to affect the CSF biomarker concentrations which argues against a general dilution effect. Interestingly, the volume of 4th ventricle was statistically significant with both L-CSF T-tau and P-Tau_181_ in patients with amyloid and tau pathologies, which somewhat supports the dilution effect. This indicates a more complex relationship between CSF biomarker levels, iNPH and AD pathology.

Decreased levels of proteins associated with neuronal plasticity and dysfunction of blood-brain barrier have been observed to affect t-Tau, amyloid-β protein precursor and amyloid-β levels in AD.^32^ This suggests that similar mechanisms could be behind decreased concentrations of CSF AD biomarkers in iNPH. Higher levels of CSF P-Tau_181_ have been associated with increased levels of neuronal pentraxin-2, which is a synaptic protein associated with neuronal plasticity. This suggests that decreased neuronal plasticity could be one factor behind lowered concentrations of tau proteins in iNPH.^33^ Our study did not investigate neuronal plasticity associated biomarkers to confirm or deny this hypothesis and this would be relevant to investigate in future studies.

We observed an inverse relation between L-CSF T-Tau and P-Tau_181_ and the volume of 4th ventricle in patients with amyloid pathology in brain biopsy. Similar observation was made in patients with Tau pathology in brain biopsy. Previously studies on CSF flow imaging have observed abnormal CSF flow and increased shear stress between 3rd and 4th ventricles.^34,35^ In vitro studies have observed that shear stress promotes fibrillization of amyloid in CSF.^36–38^ Makibatake et al. observed that Aβ_1-42_ impairs ciliary function, which impacts clearance of CSF.^39^ Ciliary function is indicated to affect the ventricular development in animal study using zebrafish larvae.^40^ Glymphatic system impairment and dysfunction of aquaporin-4 have been observed in multiple neurological disorders.^41^ In iNPH impaired ciliary function has been implicated as dysfunction of genes such as CFAP43, which is associated with ciliary function, is a potential causative gene for iNPH.^42^ Glymphatic system impairment has also been observed to be associated with amyloid pathology in PET imaging.^43^ Similar observation has also been made in mouse model regarding tau pathology.^44^ We theorize that abnormal CSF flow observed in iNPH can promote both amyloid and tau pathology via impairment of glymphatic system and that the observed negative correlation between 4th ventricle and L-CSF T-Tau and P-Tau_181_ could be a result of abnormal CSF flow and impairment of glymphatic function in iNPH patients.

Larger hippocampal volume seems to reduce the total amount of Tau in LCSF but seems to have no effect on total amount of Aβ_1-42_—as expected according to the previous studies.^45,46^ Although there is a potential genetic link between P-Tau_181_ and ventricular volume, we failed to find an association between ventricular volume and P-Tau_181_.^47^ However, this is similar to previous studies in both iNPH and AD.^21,48^

We noticed unexpected difference between Amyloid + Tau + -group and Amyloid - Tau + -group in V-CSF Aβ_1-42_ concentration (Table 1B). The group with amyloid pathology had higher levels of V-CSF Aβ_1-42_. The number of patients in these groups was low (6 and 5 respectively) so this should be interpreted cautiously. Although higher levels of Aβ_1-42_ could be explained by impaired CSF clearance.^49^

The strength of our study is in the ability to standardize the association between CSF AD-biomarker levels and ventricle volumes according to brain biopsy AD-pathology. However, the underlying reason for the generally decreased CSF AD-biomarker levels in iNPH remains unclear and requires further studies.

Our study is limited by the lack of follow-up data for the clinical diagnosis of AD, primarily due to limited follow-up time from recent shunting. Another consideration is that the nature of amyloid pathology is patchy, which may result in false negatives in the amyloid negative group, but the rate of false negatives should be low as biopsy results correlate well with Amyloid PET findings.^50^ Another limitation is that static MRI imaging cannot capture the movement of CSF and using dynamic imaging techniques would further aid in evaluating CSF flow dynamics. Although alcohol usage can potentially affect CSF AD biomarker levels, we did not include this into analyses as reporting of alcohol usage in clinical settings is inconsistent.

### Conclusion

The intracerebral ventricular volume does not dilute CSF biomarker concentrations in iNPH patients. However, an inverse relation between L-CSF T-Tau and P-Tau_181_ concentration and 4th ventricle was observed in iNPH patients with amyloid and tau pathologies, suggesting a complex relationship between AD pathology, CSF volume, and CSF flow in iNPH patients.

## Supplemental Material

sj-docx-1-alz-10.1177_13872877251329081 - Supplemental material for Effect of ventricular volume on cerebrospinal fluid Alzheimer's disease biomarkers in patients with idiopathic normal pressure hydrocephalus Supplemental material, sj-docx-1-alz-10.1177_13872877251329081 for Effect of ventricular volume on cerebrospinal fluid Alzheimer's disease biomarkers in patients with idiopathic normal pressure hydrocephalus by Aleksi Vanninen, Lauri Erkkilä, Tarja Kokkola, Tuomas Selander, Anne M Koivisto, Merja Kokki, Tadeusz Musialowicz, Anssi Lipponen, Mikko Hiltunen, Juhana Hakumäki, Sanna-Kaisa Herukka, Tuomas Rauramaa and Ville Leinonen in Journal of Alzheimer's Disease
