# Supplementary material for: Effect of ventricular volume on cerebrospinal fluid Alzheimer's disease biomarkers in patients with idiopathic normal pressure hydrocephalus
Source: J Alzheimers Dis. 2025 Mar 28;105(2):397–406. doi: 10.1177/13872877251329081 (PMC13066472; doi:10.1177/13872877251329081)
Supplement: sj-docx-1-alz-10.1177_13872877251329081 - Supplemental material for Effect of ventricular volume on cerebrospinal fluid Alzheimer's disease biomarkers in patients with idiopathic normal pressure hydrocephalus [file sj-docx-1-alz-10.1177_13872877251329081.docx]

**Supplemental Material**

**Effect of ventricular volume on cerebrospinal fluid Alzheimer’s disease biomarkers in patients with idiopathic normal pressure hydrocephalus**

**Linear regression models for non-normalized lumbar CSF values**

**Supplemental Table 1.** Linear regression models for investigated L-CSF biomarkers in relation to regional brain volumes.

| Dependent | log_10_P-Tau_181_ |  |  |
| --- | --- | --- | --- |
| Variable | **B** | **p** | **95% C.I.** |
| 4^th^ ventricle | -0.045 | 0.006 | -0.077­ — -0.013 |
| Hippocampus | -0.043 | 0.026 | -0.080 — -0.005 |
| Dependent | **log_10_T-Tau** |  |  |
| Variable | **B** | **p** | **95% C.I.** |
| 4^th^ ventricle | -0.028 | 0.005 | -0.048 — -0.009 |
| Hippocampus | -0.050 | <0.001 | -0.073 — -0.027 |
| Dependent | **log_10_Aβ_1-42_** |  |  |
| Variable | **B** | **p** | **95% C.I.** |
| 4^th^ ventricle | -0.028 | 0.019 | -0.052— -0.005 |
| Occipital pole (left) | 0.053 | 0.008 | 0.014 — 0.092 |

L-CSF: Lumbar cerebrospinal fluid; B: Regression coefficient; C.I.: confidence interval for regression coefficient; Aβ_1-42_: amyloid beta 1-42; t-Tau: total tau protein; P-Tau_181_: phosphorylated tau 181.

**Supplemental Table 2.** Linear regression models for investigated L-CSF biomarkers in relation to regional brain volumes in female subgroup.

| Dependent | log_10_P-Tau_181_ |  |  |
| --- | --- | --- | --- |
| Variable | **B** | **p** | **95% C.I.** |
| No statistically significant variables | - | - | - |
| Dependent | **log_10_T-Tau** |  |  |
| Variable | **B** | **p** | **95% C.I.** |
| 4^th^ ventricle | -0.043 | 0.007 | -0.074 — -0.012 |
| parahippocampal gyrus (right) | -0.125 | 0.002 | -0.202 — -0.047 |
| anterior orbital gyrus | 0.087 | 0.001 | 0.037 — 0.136 |
| Superior parietal lobule left | -0.025 | 0.016 | -0.044 — -0.005 |
| Dependent | **log_10_Aβ_1-42_** |  |  |
| Variable | **B** | **p** | **95% C.I.** |
| 4^th^ ventricle | 0.083 | 0.001 | -0.083 — .0.014 |
| anterior orbital gyrus | -0.048 | 0.006 | 0.035 — 0.131 |
| Caudate (left) | 0.086 | 0.015 | 0.017 — 0.154 |

L-CSF: Lumbar cerebrospinal fluid; B: Regression coefficient; C.I.: confidence interval for regression coefficient; Aβ_1-42_: amyloid beta 1-42; t-Tau: total tau protein; P-Tau_181_: phosphorylated tau 181.

**Supplemental Table 3.** Linear regression models for investigated L-CSF biomarkers in relation to regional brain volumes in male subgroup.

| Dependent | log_10_P-Tau_181_ |  |  |
| --- | --- | --- | --- |
| Variable | **B** | **p** | **95% C.I.** |
| No statistically significant variables | - | - | - |
| Dependent | **log_10_T-Tau** |  |  |
| Variable | **B** | **p** | **95% C.I.** |
| Hippocampus | -0.105 | 0.006 | -0.179 — -0.031 |
| Inner temporal lobe | 0.057 | 0.038 | 0.003 — 0.111 |
| Dependent | **log_10_Aβ_1-42_** |  |  |
| Variable | **B** | **p** | **95% C.I.** |
| No statistically significant variables | - | - | - |

L-CSF: Lumbar cerebrospinal fluid; B: Regression coefficient; C.I.: confidence interval for regression coefficient; Aβ_1-42_: amyloid beta 1-42; t-Tau: total tau protein; P-Tau_181_: phosphorylated tau 181.
